# Simultaneous Quantitative and Qualitative Analysis of Flavonoids from Ultraviolet-B Radiation in Leaves and Roots of *Scutellaria baicalensis* Georgi Using LC-UV-ESI-Q/TOF/MS

**DOI:** 10.1155/2014/643879

**Published:** 2014-03-13

**Authors:** Wen-Ting Tang, Min-Feng Fang, Xiao Liu, Ming Yue

**Affiliations:** Key Laboratory of Resource Biology and Biotechnology in Western China, Ministry of Education, The College of Life Sciences, Northwest University, No. 229 Taibai North Road, Xi'an, Shaanxi 710069, China

## Abstract

*Scutellaria baicalensis* Georgi is one of the most widely used traditional Chinese herbal medicines. It has been used for anti-inflammatory, anticancer, antibacterial activities, and so forth. Long-term enhanced ultraviolet-B (UV-B) radiation caused more effect on leaves than on roots of the plant. Liquid chromatography-ultraviolet detection coupled with electrospray ionization quadrupole time-of-flight mass spectrometry (LC-UV-ESI-Q/TOF/MS) method was applied for simultaneous quantitative and qualitative analysis of flavonoids in leaves and roots of *S. baicalensis* by enhanced UV-B radiation. Both low-intensity radiation and high-intensity radiation were not significantly increaseing the contents of baicalin, wogonoside, and wogonin in roots. However different intensity of radiation has different effects on several flavonoids in leaves. Both low-intensity radiation and high-intensity radiation had no significant effect on contents of baicalin and tectoridin in leaves; the content of scutellarin was significantly decreased by low-intensity radiation; chrysin was detected in low-intensity radiation and high-intensity radiation, and chrysin content is the highest in low-intensity radiation, but chrysin was not detected in control group. Different changes of different flavonoids under enhanced UV-B radiation indicate that induction on flavonoids is selective by enhanced UV-B radiation.

## 1. Introduction

*Scutellaria baicalensis* Georgi (Labiatae) is a kind of perennial herb. It is one of the most widely used traditional Chinese medicines and is officially listed in the Chinese Pharmacopoeia. Its roots have been used for anti-inflammation and anticancer, treating bacterial and viral infections of the respiratory and the gastrointestinal tract, cleaning away heat, moistening aridity, purging fire, detoxifying toxicosis, reducing the total cholesterol level, and decreasing blood pressures. This herb also possesses cholagogic, diuretic, and cathartic actions. Some concentrated composite herbal preparations that contain* S. baicalensis* as a major ingredient in their prescriptions are widely used in oriental countries [1, 2].

*S. baicalensis* contains a variety of flavonoids, phenylethanoids, amino acids sterols, and essential oils. Its dried roots contain over 30 kinds of flavonoids, such as baicalin, baicalein, wogonin, wogonin 7-O-glucuronide, oroxylin A, and oroxylin A 7-O-glucuronide [3]. Baicalin, baicalein, wogonin, and wogonoside are the main components and have significant pharmacological effects [4, 5]. Chemical composition of root of* S. baicalensis *has been comprehensive studies [3]. However chemical components of leaf were still inadequate.

Researchers have paid attention to ultraviolet-B (UV-B) radiation on the effects of plant secondary metabolism. It has significant effects on contents of secondary metabolites in many natural medicines. Several studies showed that contents of phenols, alkaloids, essential oil, and cannabinoids could be induced by enhanced UV-B radiation [6–9]. UV-B radiation has important effects on antioxidant systems and secondary metabolism of* S. baicalensis*. The determination of current LC-UV-ESI-Q/TOF/MS focuses on enhanced UV-B radiation effects on active ingredient contents in the leaves and roots of* S. baicalensis*. This study can understand effects of long-term enhanced UV-B radiation on contents of the flavonoids from leaves and roots of* S. baicalensis*.

## 2. Experimental

### 2.1. Chemicals and Reagents

Baicalin (number 110715-201016), tectoridin (number 111632-200501), and chrysin (number 111701-200501) were purchased from National Institute for the Control of Pharmaceutical and Biological Products; wogonoside (number E-0664), scutellarin (number E-0554), and wogonin (number E-0103) were purchased from Shanghai Tongtian Biotech Company. LC-grade acetonitrile was purchased from the Fisher Company. Ultrahigh purity water was prepared using a Milli-Q water purification system. Other chemicals were of analytical grade and their purity was above 99.5%.

### 2.2. Plant Materials and UV-B Irradiation

Seeds were bought from planting base in Shangluo, Shaanxi, in April 2009. Seeds were sowed at the Northwest University Biological Park and then routine management were conducted. Plants were randomly divided into control group (CK), low-intensity UV-B group (TL), and high-intensity UV-B group (TH) in April 19, 2010. Enhanced UV-B treatment was conducted every day from 9:00 to 17:00 for the TL and TH groups, except rainy day. The treatment was conducted until September 3, 2010. UV-B radiation was provided by UV-B Lamps (40 W, wavelength 313 nm), and the lamps were placed below the treatment group. UV-B intensities were measured using a UV radiometer intensity of UV-B radiation for TL and TH groups at the top of plants that were 12.1 *μ*W/cm and 34.5 *μ*W/cm, respectively.

### 2.3. Sample Preparation and Determination

After harvesting, seven plants CK, TL, and TH groups were dried at 105°C for 30 min and then were dried 80°C for 72 h. Roots and leaves were crushed with a muller and filtered through 40 mesh sieve. After that, they were placed in cool, dry, and dark environment. Samples were prepared following reported methods and made appropriate modification [10]. 0.1 g leaf sample and 0.05 g root sample were extracted with 50 mL solvent including methanol : water : formic acid (70 : 29 : 1) by ultrasonic method for 120 min. Extracted solutions were centrifuged for 7 min at 13000 rpm; then, the supernatant was filtered through 0.22 *μ*m filters.

### 2.4. LC-MS Condition and LC Condition

LC-UV-ESI-Q/TOF/MS analysis was carried out according to the reported method by Horvath et al. [10], with minor modifications. Analysis was performed with an UltiMate3000 liquid chromatographic system (Dionex, USA) equipped with a MacroTOF-QII (Bruker, German). The chromatographic separation was performed on a C18 analytical column (4.6 mm × 150 mm, 5 *μ*m i.d.) with the column temperature set at 30°C. The mobile phase consisted of acetonitrile (A) and formic acid water (pH = 3) (B). The optimized HPLC elution conditions were as follows: 0–5.0 min, 17–30% A; 5.0–10.0 min, 30–50% A; 10.0–15.0 min, 50–100% A; 15.0–20.0 min, 100–17% A; and 20.0–25.0 min, 17% A. The flow rate was 0.3 mL/min with detector wavelength set at 278 nm, and the injection volume was 10 *μ*L. Mass spectrometry was performed on electrospray ionization source in the negative mode.

LC-UV analysis was determined following reported methods [10], with minor modification. Analysis was performed with an UltiMate3000 liquid chromatographic system (Dionex, USA). The chromatographic separation was performed on a Hypersil ODS2 analytical column (4.6 mm × 150 mm, 5 *μ*m i.d.) with the column temperature set at 30°C. The mobile phase consisted of acetonitrile (A) and formic acid water (pH = 3) (B). The optimized LC elution conditions were as follows: 0–5.0 min, 17%–30% A; 5.0–10.0 min, 30%–50% A; 10.0%–15.0 min, 50%–100% A; 15.0–20.0 min, 100%–17% A; and 20.0–25.0 min, 17% A. The flow rate was 1.0 mL/min with detector wavelength set at 278 nm, and the injection volume was 10 *μ*L.

### 2.5. Preparation of Standard Solutions

Standard stock solutions of scutellarin (1000 *μ*g/mL), tectoridin (120 *μ*g/mL), baicalin (1120 *μ*g/mL), chrysin (1140 *μ*g/mL), wogonoside (120 *μ*g/mL), and wogonin (1160 *μ*g/mL) were prepared by dissolving suitable amounts of pure substance in methanol and were stored in darkness at 4°C. Working standard solutions containing scutellarin (100 *μ*g/mL), tectoridin (50 *μ*g/mL), baicalin (100 *μ*g/mL), chrysin (114 *μ*g/mL), wogonoside (50 *μ*g/mL), and wogonin (116 *μ*g/mL) were prepared by diluting the stock solutions with methanol to a series of proper concentrations. All the solutions were stored in the refrigerator at 4°C before analysis.

### 2.6. Calibration Curves, Correlation Coefficient, and Linear Range of Six Flavonoids

A calibration curve is used to determine the calculated concentration of the samples and triplicate injections. The curve is a plot of the standards' concentration against the area of tested compound by known concentration on the *x*-axis and the measured area on the *y*-axis. The calibration curve of each compound was performed with at least six appropriate concentrations. The linearity was evaluated by linear regression analysis calculated by the least square regression method.

### 2.7. Statistical Analysis

All experiments were performed in six times repeatedly. Statistical analyses were performed with STATISTICA 6.0. The results were expressed as the means ± standard error (S.E.) of triplicate. The data were subjected to one-way analysis of variance (ANOVA) and the significance of difference between samples means was calculated by Duncan's multiple range test and *P* values less than 0.05 were considered significant.

## 3. Results

### 3.1. Linearity, Limit of Detection, and Limit of Quantification

To improve quantification precision and repeatability, the curves of six flavonoids are plots of the standards' concentration against the area of tested flavonoids. It then applies the area of the flavonoids in real samples to the curves and finds the concentration from the *x*-axis to determine the calculated concentration. Calibration curves, correlation coefficient, and linear range of six tested flavonoids were shown in Table 1. The calibration curves generated from detection of sample containing known amounts of the six flavonoids were linear over the quantities ranges from 0.2 *μ*g/mL to 100 *μ*g/mL. The correlation coefficient (*r*) for each of these calibration curves was over 0.99, indicating a good linear detector response dynamic range that was investigated.

### 3.2. Results of LC-UV-ESI-Q/TOF/MS Analysis

**Figure 1:**
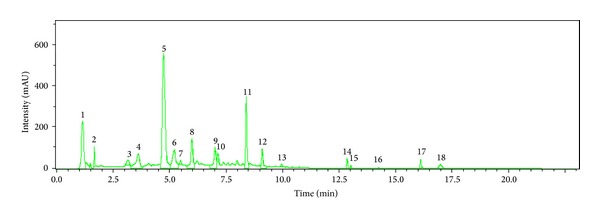
Typical chromatogram of LC-UV-MS from TL-leaves of* S. baicalensis*.

**Figure 2:**
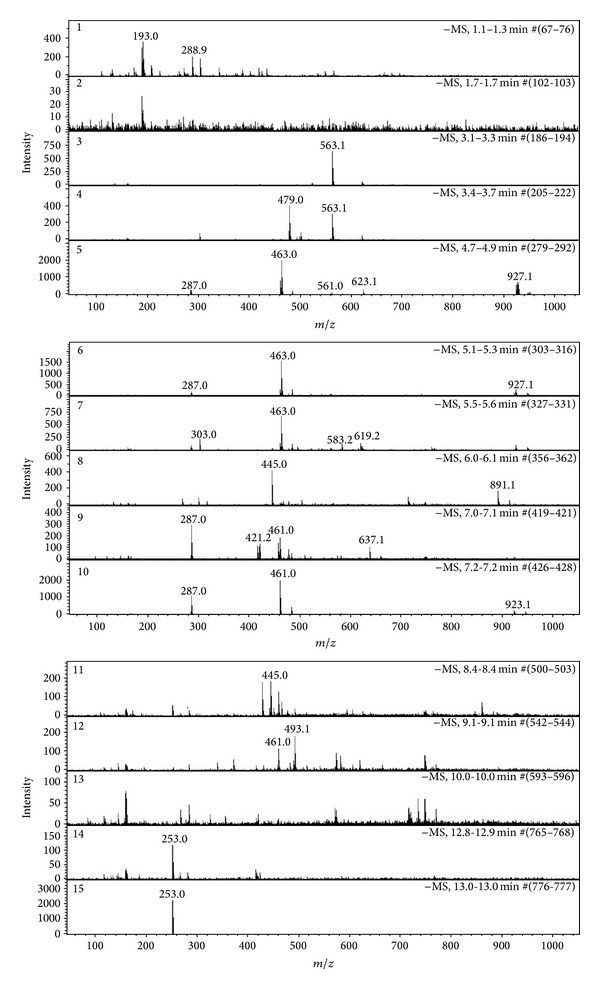
Corresponding mass spectra from HPLC-UV chromatogram in TL-leaves of* S. baicalensis*. Numbers consist with Figure 1.

LC-UV-ESI-Q/TOF/MS chromatograms and corresponding mass spectra of the TL-leaves of* S. baicalensis* were shown in Figures 1 and 2. Peaks 5 and 6 were* m/z* 463.0. It has been confirmed that scutellarin existed in leaves of* S. baicalensis* (*m/z* is 462.3).* m/z* 462.4 was fragment ion of tectoridin, which was close to scutellarin and both of the two compounds were isoflavone, so we concluded that peaks 5 and 6 were scutellarin and tectoridin, respectively. Peaks 8 and 11 were* m/z* 445.0, which suggested that the possible compositions were apigenin-7-O-glucuronide (*m/z* 446.3) and baicalin (*m/z* 446.3). Peak 9 was consistent with several peaks, in which the greatest relative abundance was* m/z* 287.0 and it was baicalein's peak (*m/z* 286.6). Peaks 14 and 15 were* m/z* 253.0 and it was chrysin's peaks.

### 3.3. Comparison of the Relative Peak Area of Flavonoids

**Figure 3:**
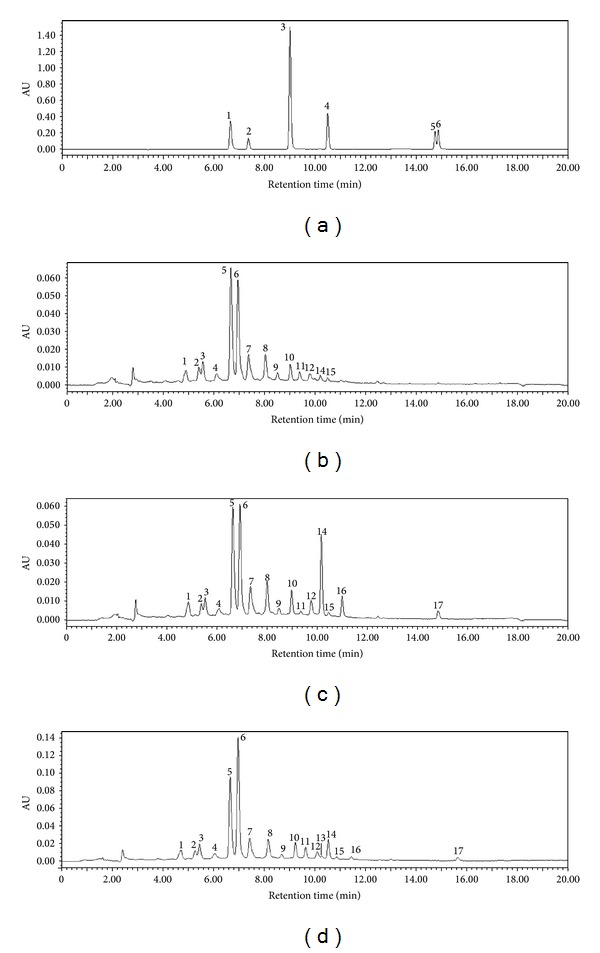
Typical chromatograms of flavonoids standards (a) and chromatograms of CK (b), TL (c), and TH (d) from leaves of* S. baicalensis*. Peaks 5, 7, 10, and 17 are the chromatogram peaks of scutellarin, tectoridin, baicalin, and chrysin, respectively. 1: scutellarin, *t*_*r*_ = 6.64 min; 2: tectoridin, *t*_*r*_ = 7.31 min; 3: baicalin, *t*_*r*_ = 9.02; 4: wogonoside, *t*_*r*_ = 10.55 min; 5: wogonin, *t*_*r*_ = 14.70 min; 6: chrysin, *t*_*r*_ = 14.81 min.

Typical chromatograms of CK, TL, and TH from leaves of* S. baicalensis* were shown in Figure 3 and the relative peak area from leaves of* S. baicalensis* was shown Table 2. Relative peak areas of peaks 6, 7, 10, and 14 were significantly increased in low-intensity UV-B radiation group and high-intensity UV-B radiation group compared with control group. Relative peak areas of peaks 1, 8, 12, and 15 were significantly increased in low-intensity UV-B radiation group compared with control group. Relative peak areas of peaks 3 and 11 were significantly increased in high-intensity UV-B radiation group compared with control group. Other relative peak area of peaks had a slight change in low-intensity UV-B radiation group and high-intensity UV-B radiation group. The relative peak area of the peak 14 produced a maximum increase by 10 times in low-intensity UV-B radiation. The relative peak area of the peak 6 produced a maximum increase by 1.4 times in high-intensity UV-B radiation. This may indicate that these types of flavonoids were induced by UV-B radiation and led to the contents increased significantly. However relative area of peak 2 was decreased in low-intensity UV-B radiation group and high-intensity UV-B radiation group. Relative peak area of the peak 4 was decreased in low-intensity UV-B radiation. Except 14 common peaks, some peaks only appeared under low- and/or high-intensity UV-B radiation. Peaks 16 and 17 were detected in two enhanced UV-B radiation groups and their relative peak areas were larger in low-intensity UV-B radiation, while peak 13 was only detected in high-intensity UV-B radiation. These results showed that enhanced UV-B radiation could increase contents of flavonoids in leaves of* S. baicalensis*. We inferred that enhanced UV-B radiation could increase content of some flavonoids in the leaves of* S. baicalensis*.

### 3.4. Quantitative Analysis of Several Flavonoids

**Figure 4:**
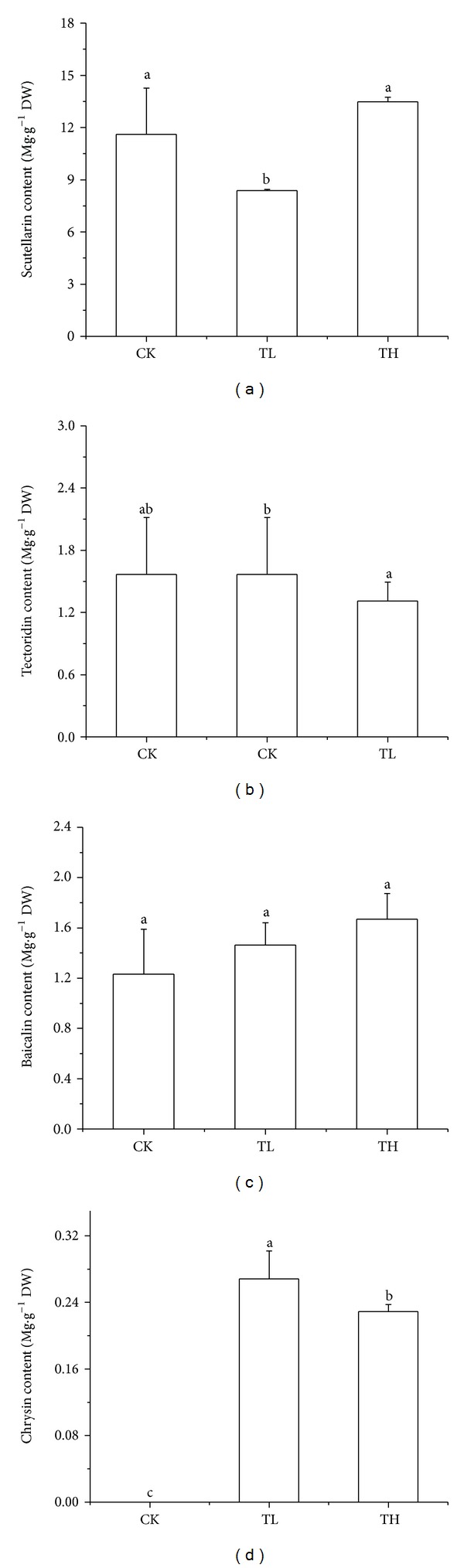
The contents of scutellarin, tectoridin, baicalin, and chrysin of CK, TL, and TH from leaves of* S. baicalensis*. Different alphabet followed the data of same index at the same treatment time that indicates significant difference among treatments.

Figure 3 showed LC of CK, TL, and TH from leaves of* S. baicalensis*. Peak 1, 2, 3, and 6 are scutellarin, tectoridin, baicalin, and chrysin, respectively.  Figure 4 showed flavonoid contents in leaves of* S. baicalensis*. It can be seen that different intensities of UV-B radiation have different effects on several flavonoids in leaves of* S. baicalensis*. Low-intensity and high-intensity UV-B radiation had no significant effects on contents of baicalin and tectoridin in leaves of* S. baicalensis*; low-intensity UV-B radiation significantly decreased the content scutellarin; chrysin was not detected in the control group while chrysin was detected in low-intensity and high-intensity UV-B radiation groups, and chrysin content is the highest in the low-intensity UV-B radiation group.

**Figure 5:**
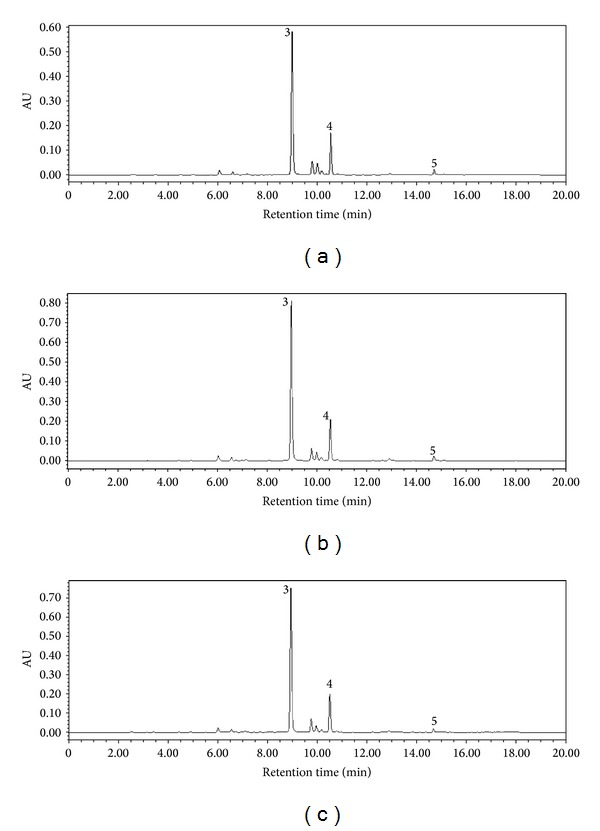
Typical chromatograms of CK (a), TL (b), and TH (c) from root of* S. baicalensis*.

**Figure 6:**
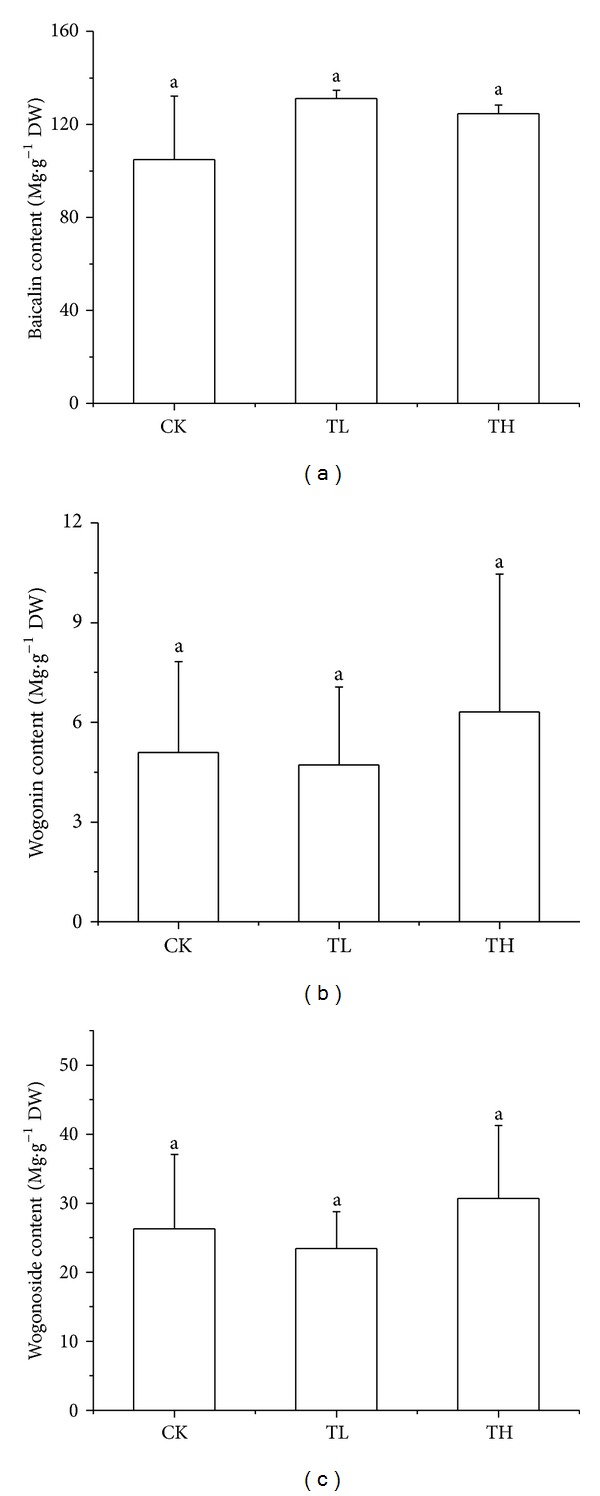
The contents of baicalin, wogonoside, and wogonin of CK, TL, and TH form roots of* S. baicalensis*. Different alphabet followed the data of same index at the same treatment time that indicates significant difference among treatments.

Figure 5 showed LC of extracts from roots of* S. baicalensis*. Peaks 3, 4, and 5 are baicalin, wogonoside, and wogonin, respectively.  Figure 6 showed flavonoid contents in roots of* S. baicalensis*. It can be seen that both the low-intensity and high-intensity enhanced UV-B radiation have not increased the contents of baicalin, wogonoside, and wogonin in root of* S. baicalensis*.

## 4. Discussions

HPLC method was used for influence of enhanced UV-B radiation on the chemical composition of plants. Flavonoids including the active ingredient of* S. baicalensis* can be accurately determinated based on this method.* S. baicalensis* was exposed under long-term UV-B radiation to understand the response of flavonoids in whole leaves and roots of plants under enhanced UV-B radiation. There is no significantly difference among the enhanced UV-B radiation group compared with control group, although baicalin content has a trend of increase under low-intensity enhanced UV-B radiation, as well as contents of wogonoside and wogonin under high-intensity enhanced UV-B radiation.

The content of scutellarin in leaves of* S. baicalensis* even decreased in low-intensity enhanced UV-B radiation. The contents of tectoridin and baicalin had no significant difference compared with the control group while they showed a trend of increase under high-intensity enhanced UV-B radiation. Chrysin was not detected in the control group, while chrysin was detected in the low-intensity and high-intensity enhanced UV-B radiation, and chrysin content was the highest under the low-intensity UV-B radiation. Results indicated that the several flavonoids were affected by different enhanced UV-B radiation, and enhanced UV-B radiation on leaves was stronger than roots. Flavonoids of relative peak area in leaves indicated significant increase of several flavonoids under enhanced UV-B radiation.

The reported literature showed the ratio of flavonoids with* ortho*-dihydroxy to those only with 4′-hydroxy in ring B of the flavonoid skeleton increase [11]. Quercetin and kaempferol were flavonoids with 3′,4′-*ortho*-hydroxy and 4′-hydroxy, respectively. The shift from kaempferol to quercetin was of interest. Increase level of quercetin was significantly higher than kaempferol under UV-B radiation [12]. It was demonstrated that quercetin showed a better scavenger of superoxide than kaempferol* in vitro* [13]. Other studies found the same shift between apigenin and luteolin derivatives [14]. Studies indicated that quercetin can more effectively scavenge hydroxyl radicals than kaempferol [15]. Olsson et al. reported that the contents of quercetin (or luteolin) derivatives had a significant increase after enhanced UV-B exposure in species capable of the shift in compound. These compounds were considered as an indicator of stress, especially if this translates into a greater capacity for antioxidative function.

Several studies have explored the mechanism of accumulation of plant secondary metabolites by enhanced UV-B radiation. Increase of some essential oil under enhanced UV-B radiation could promote development of vegetable oil glands [16]. A well-established UV-B effect thought to be mediated by the postulated UV-B photoreceptor is the induction of phenylpropanoid biosynthetic pathway components leading to the accumulation of sunscreen flavonoids [17].

## 5. Conclusions

This study is the first application of LC-UV-ESI-Q/TOF-MS method to routine identification and determination of the flavonoids from ultraviolet-B radiation in leaves and roots of* Scutellaria baicalensis* Georgi. Long-term enhanced UV-B radiation caused more effect on leaves than on roots of the plant. An amount of some flavonoids are significantly increased under low-intensity or (and) high-intensity UV-B radiation, while others do not change significantly. Different changes of different flavonoids under enhanced UV-B radiation indicated that induction on flavonoids by enhanced UV-B radiation is selective.

## Figures and Tables

**Table 1:** Calibration curves, correlation coefficient, and linear range of six flavonoids.

Analyte	Calibration curve	*r*	Linear range (*μ*g/mL)
Scutellarin	*y* = 12671 × −42837	0.9979	3.13–100
Tectoridin	*y* = 25559 × −18475	0.9957	0.75–24.0
Baicalin	*y* = 26364 × −1345	0.9973	0.36–11.40
Chrysin	*y* = 29604 × −56360	0.9982	1.75–112.0
Wogonoside	*y* = 45361 × −8145	0.9987	0.38–24.0
Wogonin	*y* = 39610 × −3237	0.9978	0.20–12.76

**Table 2:** Relative peak area of CK, TL, and TH from leaves of *S. baicalensis*.

Number	CK	TL	TH
1	0.14	0.17	0.15
2	0.14	0.13	0.12
3	0.19	0.20	0.24
4	0.17	0.14	0.17
5	1.00	1.00	1.00
6	0.90	0.96	1.30
7	0.33	0.40	0.38
8	0.31	0.39	0.33
9	0.10	0.11	0.09
10	0.16	0.31	0.22
11	0.11	0.09	0.17
12	0.09	0.18	0.11
13	#	#	0.04
14	0.06	0.61	0.21
15	0.04	0.06	0.03
16	#	0.16	0.03
17	#	0.08	0.04

(#): not detected.
